# Metacognitive Reflection and Insight Therapy (MERIT) with a Patient with Persistent Negative Symptoms

**DOI:** 10.1007/s10879-016-9333-8

**Published:** 2016-05-28

**Authors:** R. J. M. van Donkersgoed, S. de Jong, G. H. M. Pijnenborg

**Affiliations:** 1 Department of Clinical Psychology and Experimental Psychopathology, Faculty of Behavioral and Social Sciences, University of Groningen, Groningen, The Netherlands; 2 Department of Education and Research, Friesland Mental Health Care Services, Leeuwarden, The Netherlands; 3 Department of Psychotic Disorders GGZ-Drenthe, Assen, The Netherlands

**Keywords:** Metacognition, Negative symptoms, MERIT, Case study

## Abstract

Metacognition comprises a spectrum of mental activities involving thinking about thinking. Metacognitive impairments may sustain and trigger negative symptoms in people with schizophrenia. Without complex ideas of the self and others, there may be less reason to pursue goal-directed activities and less ability to construct meaning in daily activities, leading to the experience of negative symptoms. As these symptoms tend to be nonresponsive to pharmacotherapy and other kinds of treatment metacognition might be a novel treatment target; improvement of metacognition might lead to improvements in negative symptoms. One therapy that seeks to promote metacognition is the Metacognitive Reflection and Insight Therapy (MERIT). In this study, a case is presented in which a first episode patient with severe negative symptoms is treated with MERIT. A case illustration and the eight core principles of MERIT are presented. Independent assessments of metacognition and negative symptoms before and after therapy show a significant increase of metacognition and decrease of negative symptoms over the course of 40 weeks.

## Introduction

Metacognition is a psychological function that comprises a spectrum of mental activities involving thinking about thinking. It includes activities ranging from discrete acts such as recognizing thoughts and feelings to more synthetic acts, in which an array of intentions, thoughts and feelings are integrated into a larger, complex representation of the self, others and the world (Semerari et al. 2003). As a recent review shows, metacognition is linked to several factors including intrinsic motivation, experience of recovery, functional competence, stigma resistance, therapeutic alliance and psychosocial function (Lysaker and Dimaggio 2014).

In addition to these factors, metacognition has been linked to negative symptoms (Lysaker et al. 2005; Nicolo et al. 2012; Macbeth et al. 2014; Luther et al. 2016). Initially labeled “avolitional syndrome” by Kraepelin (1919), negative symptoms are defined as the absence of normal behaviors and functions. Negative symptoms such as blunted affect, avolition, anhedonia, loss of interest and paucity of thought content are associated with major negative effects on patients’ quality of life, functional status and long-term outcome (Milev et al. 2005; Kurtz et al. 2005; Möller 2007; Hunter and Barry 2012). Moreover, these symptoms often persist when positive symptoms subside and tend to be nonresponsive to pharmacotherapy (Buckley and Stahl 2007; Barnes and Paton 2011) and are therefore of particular clinical relevance. Development of effective treatment for negative symptoms remains a major challenge for the field (Buchanan 2007).

Metacognitive impairments may sustain and possibly trigger negative symptoms. A study by Lysaker et al. (2015) showed lower levels of metacognition predicted later levels of elevated negative symptoms even after controlling for initial levels of negative symptoms. Without complex ideas of the self and others, there may be less reason to pursue goal-directed activities and less ability to construct meaning in daily activities, leading to the experience of negative symptoms. Metacognition might therefore be a novel treatment target, as improvement of metacognition might lead to improvements in persistent negative symptoms and other factors in people with schizophrenia.

One therapy that seeks to promote metacognition is the Metacognitive Reflection and Insight Therapy (MERIT, in development by dr. P.H. Lysaker, see Van Donkersgoed et al., 2014). In this therapy patients are stimulated to think about their ideas of themselves and others. MERIT operationalizes metacognition as a hierarchical capacity and suggests that interventions to stimulate metacognition should be based on the patients’ current level of metacognitive functioning. Patients with lesser capacities need interventions to assist them to master basic capacities before attempting more complex ones (Lysaker et al. 2011). MERIT is built on eight core principles that the therapist has to adhere to in every session. Multiple case reports have documented the acceptability of this treatment and positive outcomes (Buck and Lysaker 2009; Lysaker et al. 2007; Salvatore et al. 2009, 2012; Hillis et al. 2015; Kukla et al. 2015; De Jong et al. 2016) and a randomized controlled trial is currently conducted in the Netherlands to assess the effectiveness of the therapy (Van Donkersgoed et al. 2014).

The present study concerns a case in which a first-episode patient with severe negative symptoms is treated with the Metacognitive Reflection and Insight Therapy. First, a case illustration is presented, followed by a description of the course of the eight core principles of MERIT in the sessions. Assessments of metacognition and negative symptoms before and after therapy will be discussed as well.

## Case Illustration

Ann is a woman in her early 20s who was raised by her parents in a town in the north of the Netherlands. Her parents moved from Italy to the Netherlands a few years before Ann was born. They had a restaurant in which they invested a significant portion of their time, from an early age their children were expected to help in the kitchen and service. Ann describes her parents as silent, hard workers. Feelings and thoughts were not discussed in the family. Her parents were strict and Ann could not remember to have ever contradicted them.

Ann’s older brother and sister both did well in school, had a large social network and did not experience any physical or mental problems. Ann, on the other hand, suffered from hearing difficulties and ear infections from an early age, which caused frequent stays in the hospital. This resulted in high absence from school and consequently in problems connecting with classmates. Her grades, however, did not suffer. Ann remembers that although she was never bullied she did not belong to any group and usually felt like an outsider. She kept to herself during breaks, reading books. When other kids tried to engage her she was shy and anxious about potentially saying or doing something wrong.

In high school she made a few friends but still kept to herself. In the beginning of her graduation year she began to experience psychotic symptoms. She began to think that her classmates and people at her school were talking about her, criticizing her and her choice of clothes. She heard them talk everywhere and even heard their voices whispering from the other side of the room. She felt like everybody was watching her and judging her. She began experiencing auditory hallucinations; voices which would criticize her constantly. In the exam period she was admitted to a hospital at her parents’ initiative, where she was diagnosed with psychosis and received atypical antipsychotic medication. After a few months of recovery she was transferred to a sheltered home for young people who had experienced their first psychotic episode. She received Cognitive Behavioral Therapy and Mindfulness-based treatment and learned practical skills for living on her own, such as cooking and cleaning. After 3 years she was transferred to a protected living flat where she could practice her newly learned skills in a more independent setting before moving to an apartment of her own.

## Negative Symptoms and Metacognitive Function Before the Start of MERIT

At the start of MERIT Ann had been living in the flat for almost a year. With help from her case manager and parents she had managed to finish the highest level of high school in the Dutch system and was now doing volunteer work in addition to helping her parents in their restaurant during the weekends. She used a regular dose of atypical antipsychotic medication and did not receive any treatment besides an occasional visit from her case manager. Every now and then she still heard voices that talked down on her, but she was able to ignore them. She no longer had the idea that everybody was talking about her. Whereas the positive symptoms were reduced, the negative symptoms had progressed: she felt empty and experienced long moments in which she did not think or feel anything. She suffered from severe avolition; while she was able to stick to a daily program developed for her by her case manager, she was unable to motivate herself without guidance from others and found herself sitting in her apartment, doing nothing at all. She was socially withdrawn and did not see others unless it was on their initiative, even though she did report that she wished she had some friends and preferred not to be alone all day. She found it difficult to engage in spontaneous conversation, often unsure what to say or ask and as a result conversations usually ended quickly. As a participant in the MERIT randomized controlled trial (Van Donkersgoed et al. 2014), Ann completed a test battery administered by an independent assessor blind to treatment condition (MERIT or treatment as usual). Negative symptoms were measured using the Positive and Negative Syndrome Scale (PANSS; Kay et al. 1987). In order to assess metacognition, a spontaneous speech sample was derived from an interview addressing the life story, the Indiana Psychiatric Illness Interview (IPII; Lysaker et al. 2010). This interview was transcribed and used by an independent, blinded consensus group to rate metacognitive performance using the Metacognition Assessment Scale—A (MAS-A, Lysaker et al. 2005).

This scale assesses the performance of the patient on four subscales of metacognition: Self reflectivity (understanding one’s own mental states), Understanding the Mind of the Other, Decentration (the ability to see the world as existing with others who have independent motives) and Mastery (the ability to use knowledge about the mental state of self and others to identify and cope with psychological problems). Pre- and post-therapy scores are presented in Tables 1 and 2 in the “Outcome” section on page 15.

Her scores on metacognitive capacity indicated that Ann, although she did not have many thoughts, was able to recognize that her thoughts were her own and she was able to distinguish between different cognitive processes in her mind, such as hoping, deciding and remembering. However, she was unable to form a nuanced idea about her own emotions and did not recognize that the ideas she had about herself and the world could change over time. She was not able to connect different thoughts and feelings in the moment or over time. Concerning other people she was able to recognize that they had thoughts that were their own, but she had difficulty distinguishing between the different cognitive processes of others. She had no idea how other people were feeling and did not seem able to form a complex picture of the internal world of others in her mind. Her score on the Decentration scale indicated that Ann was able to recognize that other people led different lives independent from her and each other, but she was not able to see that people could understand the same situation in different ways and that there are multiple ways of interpreting the same situation. Regarding Mastery, Ann was able to recognize plausible psychological problems: she expressed, for instance, that she wished she could take more initiative in her life. She saw little other options in dealing with problems than calling her mother or sister and asking them what to do. This put a strain on her relationships: on some occasions she would call her mother more than five times a day for support.

## Course of Treatment

### Element 1: The Preeminent Role of the Patient’s Agenda

The first element of MERIT asks the therapist to attend to the patient’s wishes, hopes and desires that she brings to the session, referred to as the patient’s agenda. This agenda may change or evolve during the session. The goal of this element is for the patient to become more aware of her wishes and intentions.

When asked what she wanted to talk about in the first session Ann answered with ‘I don’t know.’ She seemed to depend on the therapist to guide the conversation and got uncomfortable during silent moments. When the therapist asked what she wanted to achieve in therapy, she answered that she hoped that it would help her improve, to be ‘more normal, like everybody else’. She seemed to expect the therapist to tell her what to improve so as to become ‘more normal’. Ann automatically put herself in the subordinate, dependent position and seemed to put the therapist in the role of the professional. The therapist felt a strong appeal to determine what to talk about, to ask enough questions as to avoid uncomfortable silences and a general need of the patient to guide her into talking. In the first sessions the therapist struggled with this appeal, wanting to make Ann feel at ease by guiding her to a topic they could discuss, putting her in a position where she could answer all questions willingly. Although this made Ann feel comfortable, the therapist felt like their conversations remained within the socially acceptable realm and no substantial topics regarding what was on Ann’s mind were discussed. Instead, Ann only discussed the topics that the therapist suggested. The therapist opted to use this process as a basis for reflection, asking in the following session: ‘What do you want to talk about?’ and Ann would reply with: ‘I don’t know’. The therapist reflected on this by stating: ‘You don’t know what to talk about. You want me to determine what to talk about.’ Ann acknowledged that. They would stay silent for a while and this seemed to make Ann uncomfortable. The therapist reflected on this: ‘It is silent in your mind, you don’t know what to talk about and I am not deciding what to talk about either and this makes you uncomfortable.’ ‘Yes,’ Ann replied, ‘Silence… I don’t know what the right topic is. What do you think I should talk about?’ The therapist replied: ‘I don’t know what the right topic is; I don’t know you very well… and I can’t decide what’s important to you, so I would really like you to decide what to talk about today. For me, it doesn’t matter what the topic is, any topic is fine. And it doesn’t matter to me if it’s silent for a while, when you think about what to talk about. I don’t mind silence at all.’ This seemed to put Ann at ease. After a while she hesitantly explained that she felt like her problems would seem stupid to the therapist and that is was safer for her to follow the lead of the therapist, which turned into a conversation about Ann’s real agenda: avoiding getting criticized by the therapist.

After this, the therapist would start each session with the same question: ‘What do you want to talk about?’ In the beginning Ann would still sometimes struggle with this and they would reflect on her agenda: not wanting to do the wrong thing and get criticized. After about fifteen sessions she seemed to be more relaxed and open, and after 25 sessions she was completely used to setting the topic of conversation. When the therapist asked ‘what do you want to talk about?’ She would laugh and say: ‘I knew you were going to ask that!’ And start with what was on her mind, allowing for her and the therapist to become more aware of her wishes and intentions.

### Element 2: The Introduction of the Therapist’s Thoughts

The second element of MERIT requires the therapist to share her thoughts about the patient’s behaviors and mental activities without overriding the patient’s agenda. The goal is to help the patient develop a greater awareness of the mental states of the therapist. To make sure that there was enough space for the thoughts and agenda of the client, the therapist tried to refrain from inserting her thoughts in the beginning of the therapy—as discussed under “element 1” this was a learning process for the therapist in the interaction with Ann. She tried to make sure only to share her thoughts when it would encourage Ann to speak her own mind more and to create a safe environment in which Ann would feel free to explore and express her own thoughts and feelings. Without being aware of it, Ann appeared to fill in what she thought the therapist was thinking, which influenced the way she talked about herself. For example, in one session she talked about how it was hard for her to entertain herself when she did not have any specific activities planned for herself, on a given day. (A) ‘Then I just sit there and I have all these hours ahead of me and I just don’t know what to do… I don’t know how to start… But I shouldn’t complain, right. No, I should just get up and do something. Yes.’ (T) ‘Do you think that is what I’m thinking right now? That you shouldn’t complain and should get up and do something in that situation?’ (A) ‘Yes. I am complaining a lot and not doing anything.’ (T) ‘Actually, that is not what I was thinking at all. I was thinking that it must be very hard for you to be in that situation, not knowing how to start anything and feeling so lost.’ This led to an interaction in which Ann said that she indeed had filled in the thoughts of the therapist with what her mother usually says (‘stop complaining, start doing something’), and discovered that this assumption of the thoughts of the therapist had been incorrect.

These kinds of interactions progressed during the sessions and after a while Ann became more aware of her tendency to fill in the thoughts of the therapist. She started checking her assumptions about the thoughts of other people more often and this made her increasingly aware that these assumptions were not always right and that there were multiple things that people could think in any given situation.

### Element 3: The Narrative Episode

The third element requires the therapist to elicit narrative episodes. These narratives may pertain to any moment of the client’s life, as long as he or she is the main actor in the story told. This is done to ensure that conversations stay connected to the client’s experience and do not get too abstract. Additionally, this helps the client to connect thoughts and feelings over time. This was an easy task with Ann. Once the agenda had been established (the initial struggle), Ann was able to think and collect memories of several episodes that were connected to her agenda. For example, one session she told the therapist that she had felt like an outsider at a family party that week. Together, the therapist and Ann reflected on the entire series of events: how these events began and progressed, who she met, and what she thought and felt in those moments. She took her time to explore how she had experienced that situation and became more aware of her thoughts and feelings at the party. Together they would try to connect the situations over time and to see how her thoughts and feelings where connected by discussing earlier social situations in which she had felt like an outsider. The idea of being disconnected from others remained a recurring theme in therapy. By the last session she was able to integrate the discussed narrative episodes into a detailed story. It included her growing up with hearing problems which made her miss parts of school and led to various situations in primary school where the other children forgot to include her or ignored her. This made her feel like she was boring and made her retract from social situations. In high school she remained shy and withdrawn and worried about social acceptance. She also discussed a few instances in which interactions with her bigger sister and brother, who both made friends easily, would make her feel socially inadequate. After a period of hospitalization, the few friends she did have made no efforts to remain in contact. This reinforced the belief that she was boring and other people were not interested in her. This was strongly emphasized by the voices she heard in her head. She alienated further from other people and was unable to take any initiative at all in social situations. She was convinced that she was not able to sustain friendships, as she never had experienced long term relationships in her life. By the end of therapy she realized that she had automatically assumed that this was caused by her being boring, but she now began to realize that her hearing problems and school absence in her youth, the psychotic episode and the negative thoughts about herself had played a major role in her difficulties forming and maintaining friendships. She realized that her idea that others would not want to spend time with her made her retract from social situations, which in turn led to others not spending time with her. They discussed several strategies to deal with this vicious cycle (see “element 8, stimulating mastery”).

### Element 4: The Psychological Problem

For the fourth element of MERIT, the therapist attends to the psychological and social challenges the participant faces, to achieve a greater awareness of these challenges. This often emerged naturally after clarifying Ann’s agenda and discussing narrative episodes. Ann was already able to identify and name psychological problems at the beginning of therapy, which made this element an easy task for the therapist. For example, in the fourth session, after talking about a narrative episode in which she had been alone, she stated: ‘I wish I was able to entertain myself… I did not know what to do…’ This led to them identifying the psychological problem: feeling unable to initiate anything, which led to thoughts of inadequacy. Ann and the therapist would usually take a moment to identify the central psychological problem after discussing a narrative episode. Several other psychological problems (the thought of being boring, difficulties connecting to other people) reoccurred over time in the sessions. As time progressed Ann was increasingly able to recognize these themes in her agenda and her narrative episodes. She was able to use this awareness to consider possible solutions to deal with these problems (Element 8).

### Element 5: Reflecting on Interpersonal Processes

The fifth element of MERIT requires the therapist to address the interpersonal processes that occurred in the session, as thinking is always happening in an interpersonal context, which can be reflected upon. This is done to help the patient develop a greater awareness of how they are relating to others. As discussed in “Element 1”, Ann tended to put the therapist in the position of the professional and put herself in a subordinate position. The therapist struggled regularly with this, feeling a need to ‘rescue’ Ann and fix her problems for her by telling her what to do and to talk about. After the first sessions she became more aware of this pitfall but this awareness did not make her immune to this pitfall in subsequent sessions. Sometimes she would determine the topic of conversation again for Ann, to avoid uncomfortable silences and to protect Ann from feeling bad. Usually the therapist would become aware of this later in the session when she would sense that they were not talking about Ann’s real agenda. This was discouraging at times, as the therapist felt like she had failed again and wished she could stay out of this pitfall altogether. She tried to stay aware of this dynamic and over the course of the therapy Ann and the therapist where able to reflect on their relationship more openly. Ann became more aware of her tendency to put herself in a subordinate position and started to see this pattern in her other relationships as well—especially with her parents.

### Element 6: Reflecting on Progress

To reflect on the experience of the session by the client and to make sure progress is achieved, MERIT encourages the therapist to investigate how the client has experienced the session. By the end of each session the therapist asked Ann how she felt about the session. In the beginning it seemed hard for Ann to give her honest opinion and she would automatically state that she was happy with the conversation and the therapist. They talked about this a few times in which the therapist encouraged her to think about the session for a moment before directly stating that everything had been perfect and to express it whenever she was not happy about the session. They discussed Ann’s fear of the therapist getting mad at her when she would express criticism and the therapist reassured her that this would not happen, that her goal was to work together in the best way possible, which required Ann’s honest opinion. After about 20 sessions Ann began to cautiously express when she was not happy about something that occurred. For example, the therapist made an incorrect reflection about Ann’s thoughts in one of the last sessions. This disrupted the conversation and caused Ann to close up. At the end of the session, when the therapist asked how Ann felt the session had progressed, she reluctantly said that she had felt in that moment like the therapist did not understand her and that it had disappointed her, triggering thoughts about nobody understanding her and her being alone. The therapist responded that she thought it was very good of Ann to mention this. She apologized for the misplaced reflection and said that she tried her best, but that she sometimes misunderstood and that it would help her if Ann would correct her. Ann seemed relieved that the therapist was not angry by her expressing some criticism. This seemed to encourage her to express critical thoughts more easily in the last couple of sessions, which improved the flow of conversation as Ann would not close off when the therapist misunderstood her, but would address the miscommunication which then became the topic of conversation.

### Element 7: Stimulating Self-reflectivity and Awareness of the Others’ Mind

The seventh element of MERIT requires the therapist to offer interventions that stimulate self-reflectivity on or slightly above the level of functioning of the client. This is done to engage the patient in forming increasingly complex thoughts about herself and others.

In the beginning of therapy Ann was able to recognize different thoughts but she couldn’t recognize her feelings. She would sometimes report being ‘uncomfortable’ or how she did ‘not feel good’. The therapist would offer reflections to direct Ann to notice and name her different emotions in the narrative episodes and in the moment: ‘You felt very sad.’ ‘What did you feel exactly? Was it sadness, or anger? Or both?’ ‘It seems like you feel ashamed about not having much thoughts today.’ Ann started to name her emotions more and more, and appeared to have developed a clearer and more nuanced idea about her emotions. The therapist then started to offer Ann reflections that stimulated her to see that her thoughts changed over time and later that her wishes and intentions did not always play out in reality. By the ending of therapy the therapist offered reflections to stimulate Ann to connect different thoughts and feelings in the moment and over time. Ann made significant progress over forty sessions, although this progress was not linear: every now and then Ann would be unaware or confused about her emotions again. While this stair-step pattern of improvement (improvement—decline—improvement) appears common during metacognitive psychotherapy (Lysaker et al. 2007), the therapist struggled with discouragement, and in these moments she would sometimes make the mistake of making higher reflections that did not match Ann’s level of metacognitive functioning. This usually resulted in confusion and Ann feeling misunderstood by the therapist. This motivated the therapist to keep adjusting her reflections to the level Ann was functioning at, even when she seemed to have fallen back a few steps on the scale, as she experienced that this led to more progression than clinging to the higher level of metacognition Ann was functioning at a week before.

In the beginning of therapy Ann was aware that other people had thoughts but she was not able to differentiate between different cognitive operations of others, such as hoping, wishing and remembering. As Ann was afraid to be rejected by others and lost track of her own mind when thinking about the mind of others, the therapist refrained from reflecting on the minds of others in order to get more awareness for Ann’s own mind. After a while she started to ask Ann what she thought the therapist was thinking in the moment and sometimes asked what she thought others were thinking, hoping and wishing in narrative episodes. Ann became more aware of her tendency to think that other people had negative thoughts about her (see Element 2 and 3) but it remained difficult for her to get a detailed picture of the thoughts of others in the moment by the ending of therapy.

### Element 8: Stimulating Mastery

The eighth element of MERIT calls for the therapist to stimulate the ability to use metacognitive knowledge about self and others to frame and cope with psychological problems. By the beginning of therapy Ann was able to recognize problems and usually solved them by calling her mother or sister for support. When she became more aware of the fact that her thoughts could change over time and that there where different ways to perceive a situation, she naturally progressed to solving problems by taking a different perspective on it. The therapist tried to stimulate this by asking questions like: ‘Is there another way to look at this problem?’ ‘What could you say to yourself to feel a bit better?’ ‘How can you help yourself to keep this positive perspective?’

For example, Ann started to realize that her view of herself as being boring and uninteresting was interfering with her engaging in social situations. She started to try and take a different perspective on herself. On her own initiative she made a list of things that were interesting about her and tried to read this list every day. This helped her to engage in more social situations. The thought of being boring still reappeared but she was now more aware of this thought, she did not necessarily see it as the truth anymore and was sometimes able to take a different view.

Ann also started to recognize that she thought that silence in a conversation meant that she was boring and that this made her freeze up in conversations. To deal with this she would tell herself that she was not the only one responsible for the flow of conversation and that silences sometimes naturally occur in conversations. This helped her put less pressure on herself, after which she was able to relax more and keep thinking instead of freezing up, which made the conversation flow more naturally.

## Outcome

There were no changes in medication over the course of the therapy and Ann did not receive any additional treatment other than a visit from her case manager once every 2 weeks. After the fortieth session of MERIT, an independent assessor blind to treatment condition (MERIT vs. TAU) conducted the PANSS assessment and the IPII interview. An independent consensus group, also blind to treatment condition scored the transcript of the IPII on the four scales of metacognition, using the MAS-A. Scores are presented in Tables 1 and 2.

**Table 1:** Metacognitive performance before and after therapy

MAS-A scale	Baseline	After 40 sessions	(Min–max)
Self-reflection	3	6	(0–9)
Understanding the other	2.5	2.5	(0–7)
Decentration	1	2	(0–3)
Mastery	4	6	(0–9)

**Table 2:** Negative symptom scores on the PANSS before and after therapy

Negative symptoms	Baseline (scale: 1–7)	After 40 sessions
Blunted affect	5	2
Emotional withdrawal	4	2
Poor rapport	3	3
Passive/apathetic social withdrawal	4	3
Difficulty in abstract thinking	2	1
Lack of spontaneity and flow of conversation	3	2
Stereotyped thinking	2	2

Metacognition seems to have improved on the MAS subscales Self-reflection, Decentration and Mastery scales. Whereas Ann was initially only able to recognize her own thoughts, she was at the end of therapy capable of recognizing a wide spectrum of emotions within herself. She was also able to see that her thoughts were subjective and could change over time. She now also recognized that her wishes and intentions would not always work out as she wanted to. By the ending of therapy Ann was able to see that people could understand the same situation in different ways and that there are multiple ways of interpreting the same situation (Decentration). In a practical sense the most important improvement was on the Mastery scale; where she used to call her mother to deal with all her psychological problems, she was now able to change her own thoughts so as to deal with these problems.

This made her call her mother less and gave her greater independence, which improved their relationship and promoted Ann’s self-confidence. Ann reported feeling better now that she was able to deal with problems by taking a different perspective. However she did not improve on the Understanding the Other scale, which indicated that she still struggled with making a complex representation of the thoughts and feeling of others in her head. She did understand that the thoughts of others were their own and tried to differentiate between different cognitive operations such as hoping, wishing and dreaming in others, but this remained unclear for her most of the time. Patients typically develop self-reflection before they are able to reflect on others, as the self functions as a model for creating ideas about what is occurring in the consciousness of the other (Dimaggio et al. 2008). In other words; you need to be aware of thoughts and feelings in your own mind, before you can recognize them in others. When the capacity for self-reflection is further developed, the therapist can shift the emphasis, in future sessions, towards reflections on what others might be thinking and feeling in the narrative episodes surrounding the emerging psychological problems. Together they could explore the minds of others as to assist the patient in forming a richer understanding of the experience of others. Reflections made by the therapist, in the moment, detailing what the therapist thinks and feels can help the client further increase this understanding.

After 40 sessions, negative symptoms had decreased as shown by the PANSS scores (Table 2) and as reported by Ann. She felt less empty, reported having more thoughts and feelings when she was alone, and was more aware of these thoughts and feelings. She still needed her case manager to help her by putting structure in her days, but she was able to take some initiative in the blank spaces in the program. She had put together a list of things she liked to do and when she was bored she would pick something from this list and start doing it. She was also more able to accept it when she would not get to do something, this was not affecting her self-esteem that much anymore; she would just try to enjoy doing nothing for a moment and would tell herself that it is okay just to sit and relax (Mastery; changing her thoughts to deal with this psychological problem). By the ending of therapy she was practicing with taking initiative in relationships. She had invited some of her old housemates at the rehabilitation center to her apartment and reconnected with an old friend from school. This was difficult for her, as contact with others still regularly triggered thoughts about being boring and uninteresting, but she was now aware of these thoughts and tried not to react to them. She tried to connect to others in spite of these thoughts, hoping this would give her correcting experiences with others and eventually would desensitize the strong beliefs. She still had difficulty with spontaneous conversation but she was able to endure silences more and tried to put less pressure on herself, which helped her to not freeze up as much as she used to. This also led to some correcting experiences in which she succeeded to make conversation in a more relaxed way.

These improvements of negative symptoms may be a result of the improvements in metacognition; Ann gained a better sense and awareness of her own internal states and this seemed to lead to a richer internal experience (she reported having more thoughts and feelings than before therapy). With a more complex representation of herself and a better understanding that there are multiple ways to interpret a social situation, Ann could make more sense of social interactions and her own thoughts and emotions in these situations. This increased awareness enabled her to adjust her behavior flexibly, leading to more preferable social scenarios than before (e.g. inviting others over instead of retracting, starting a conversation with others).

## Limitations

There are limitations to this case study. It describes what happens when MERIT is used to treat a first episode, intelligent patient with negative symptoms. As with all case studies, it is not possible to generalize these results to groups of patients. It is unclear if MERIT is effective for patients with prolonged schizophrenia, lower level of education or different types or patterns of symptoms. It is also unclear if the chosen forty sessions are necessary or enough for other patients. More case studies as well as controlled trials are needed on MERIT, metacognition, its relationship with negative symptoms and ways to improve metacognition and negative symptoms in patients with schizophrenia.

## Clinical Practices and Summary

In this case report the eight elements of MERIT were used to improve metacognition in a patient with severe negative symptoms. Several observations were made concerning the implications of this study in clinical work. It suggests that it is possible to improve metacognition in therapy and that this can co-occur with a decrease of negative symptoms. It also suggests that metacognition and negative symptoms are interrelated and that negative symptoms can be understood and improved using psychotherapy. As most research efforts are concentrated on understanding the neurological basis and treating negative symptoms with antipsychotic medication or behavioral activation (Mairs et al. 2011; Tsapakis et al. 2015; Fusar-Poli et al. 2015), it is important to note that our study suggests that it may be equally important to pay attention to the psychological factors that are connected to negative symptoms and ways to improve these symptoms by improving psychological processes such as metacognition.
